# C-Nap1 mutation affects centriole cohesion and is associated with a Seckel-like syndrome in cattle

**DOI:** 10.1038/ncomms7894

**Published:** 2015-04-23

**Authors:** Sandrine Floriot, Christine Vesque, Sabrina Rodriguez, Florence Bourgain-Guglielmetti, Anthi Karaiskou, Mathieu Gautier, Amandine Duchesne, Sarah Barbey, Sébastien Fritz, Alexandre Vasilescu, Maud Bertaud, Mohammed Moudjou, Sophie Halliez, Valérie Cormier-Daire, Joyce E.L. Hokayem, Erich A. Nigg, Luc Manciaux, Raphaël Guatteo, Nora Cesbron, Geraldine Toutirais, André Eggen, Sylvie Schneider-Maunoury, Didier Boichard, Joelle Sobczak-Thépot, Laurent Schibler

**Affiliations:** 1 Institut National de la Recherche Agronomique (INRA), Unité Mixte de Recherche 1313—Génétique Animale et Biologie Intégrative (UMR1313—GABI), F-78352 Jouy-en-Josas, France; 2 Sorbonne Universités, UPMC University Paris 06, Institut de Biologie Paris Seine (IBPS)—Biologie du Développement, UMR 7622, F-75005 Paris, France; 3 CNRS, UMR 7622, IBPS—Biologie du Développement, F-75005 Paris, France; 4 INSERM, ERL 1156, F-75005 Paris, France; 5 Plate-forme SIGENAE/Génétique Cellulaire, INRA, Castanet-Tolosan F-31326, France; 6 INSERM, UMR_S 938, Saint-Antoine Research Center, F-75012 Paris, France; 7 Centre de Biologie pour la gestion des populations (CBGP)/INRA, UMR1031, F-34988 Montferrier-sur-Lez, France; 8 INRA, Unité Expérimentale (UE0326), F-61310 LE PIN-AU-HARAS, France; 9 Allice, Département R&D, F-75595 Paris, France; 10 Labogena, F-78352 Jouy-en-Josas, France; 11 INRA, Unité de Virologie et Immunologie Moléculaires (UR0892), F-78352 Jouy-en-Josas, France; 12 Institut National de la Santé et de la Recherche Médicale (INSERM), Unité U1163, Université Paris Descartes-Sorbonne Paris Cité, Département de génétique, Institut Imagine, Hôpital Necker Enfants Malades, F-75015 Paris, France; 13 Department Biozentrum, University of Basel, Klingelbergstrasse 50/70, CH-4056 Basel, Switzerland; 14 CEIA du Doubs et du Territoire de Belfort, F-25640 Roulans, France; 15 LUNAM Université, Oniris, UMR BioEpAR, CS 40706, F-44307 Nantes, France; 16 INRA, UMR1300 BioEpAR, CS 40706, F-44307 Nantes, France; 17 Sorbonne Universités, UPMC University Paris 06, Paris Seine Biology Institute, Electron Microscopy Facility, F-75005 Paris, France

## Abstract

Caprine-like Generalized Hypoplasia Syndrome (SHGC) is an autosomal-recessive disorder in Montbéliarde cattle. Affected animals present a wide range of clinical features that include the following: delayed development with low birth weight, hind limb muscular hypoplasia, caprine-like thin head and partial coat depigmentation. Here we show that SHGC is caused by a truncating mutation in the *CEP250* gene that encodes the centrosomal protein C-Nap1. This mutation results in centrosome splitting, which neither affects centriole ultrastructure and duplication in dividing cells nor centriole function in cilium assembly and mitotic spindle organization. Loss of C-Nap1-mediated centriole cohesion leads to an altered cell migration phenotype. This discovery extends the range of loci that constitute the spectrum of autosomal primary recessive microcephaly (MCPH) and Seckel-like syndromes.

## Abstract (web summary)

SHGC syndrome affects cattle and has clinical features similar to human Seckel syndrome. Here Floriot *et al*. identify the causative mutation in the centrosomal protein C-Nap1 that affects centriole cohesion and cell migration, extending the range of loci involved in human Seckel-like syndromes.

## Graphical abstract

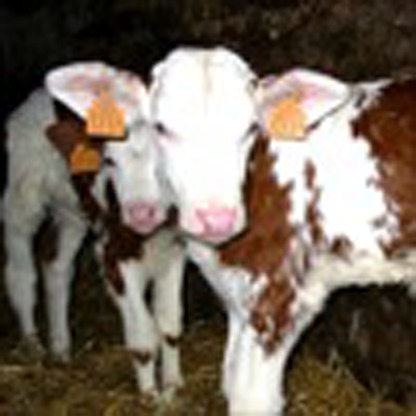

The centrosome is a major microtubule-organizing centre^1^,^2^. It is formed by a pair of centrioles surrounded by the pericentriolar material, which contains proteins responsible for microtubule nucleation and anchoring. During cell cycle progression, the two centrioles duplicate once, thereby assembling two centrosomes. The two parental centrioles are loosely connected by fibrous protein structures^3^,^4^, C-Nap1 and rootletin, along with other proteins, being the key components of this tether^5^,^6^,^7^,^8^. The C-Nap1/rootletin protein fibres are associated with the proximal ends of the centrioles through CEP135, which acts as a docking site for C-Nap1 (ref. 9). Centrosomes remain interconnected until the G2 phase, when phosphorylation of C-Nap1 by Nek2 kinase induces their separation^5^,^10^ in preparation for bipolar spindle assembly^11^. In addition, the fully mature mother centriole also functions as a basal body to assemble a primary cilium, particularly in quiescent cells. Experimental inactivation of proteins participating in centriole-centriole cohesion induces centrosome splitting independently of the cell cycle phase. Whether cohesion of the two centrioles is dispensable for centriole biogenesis and cilium assembly is unclear. While centrosomes are not essential for mitosis, they increase the efficiency of mitotic spindle assembly and are involved in proper chromosome segregation and cell division, cell adhesion, polarity and motility^12^, as well as in signalling pathways involving primary cilia^13^. Consequently, centrosome defects are associated with diverse phenotypes^1^,^14^. In humans, mutations in several centrosomal proteins, such as PCTN, STIL, CEP152, CEP135, CEP63 and CENPJ, have been associated with autosomal primary recessive microcephaly (MCPH), Majewski Osteodysplastic Primordial Dwarfism type II and Seckel syndrome^15^,^16^,^17^,^18^,^19^,^20^,^21^.

Caprine-like Generalized Hypoplasia Syndrome (or SHGC) is an autosomal recessive disorder described in the Montbéliarde cattle breed. We previously reported the characterization of this disease and mapped SHGC to a 6-Mb region on bovine chromosome 13 (ref. 22). The disease presents with a wide range of clinical features and associates muscular hypoplasia with features from the Seckel syndrome and autosomal MCPH^23^, such as delayed development, short stature, long and thin head, as well as phenotypic characteristics of neurocristopathies^24^ such as partial coat depigmentation (Fig. 1a). Using homozygosity mapping and high-throughput sequencing, we demonstrate that SHGC is caused by a truncating mutation in the *CEP250* gene that encodes the centrosomal protein C-Nap1 (ref. 5). The spontaneous mutation of *CEP250* in a cattle breed offers, thus, an opportunity to investigate *in vivo* the functions of C-Nap1. We show that SHGC mutation results in centrosome splitting and loss of the rootletin linker. Ultrastructure of mutant centrioles is not altered and the lack of centriole–centriole cohesion neither affects centriole duplication during the cell cycle nor centriole functions in cilium assembly and mitotic spindle organization. However, cell migration behaviour is altered in primary mutant fibroblasts. In conclusion, loss of C-Nap1-mediated centriole cohesion leads to a phenotype that extends the range of loci constituting the spectrum of autosomal MCPH and Seckel-like syndromes.

## Results and Discussion

### SHGC is linked to a CEP250 gene mutation

**Figure 1:**
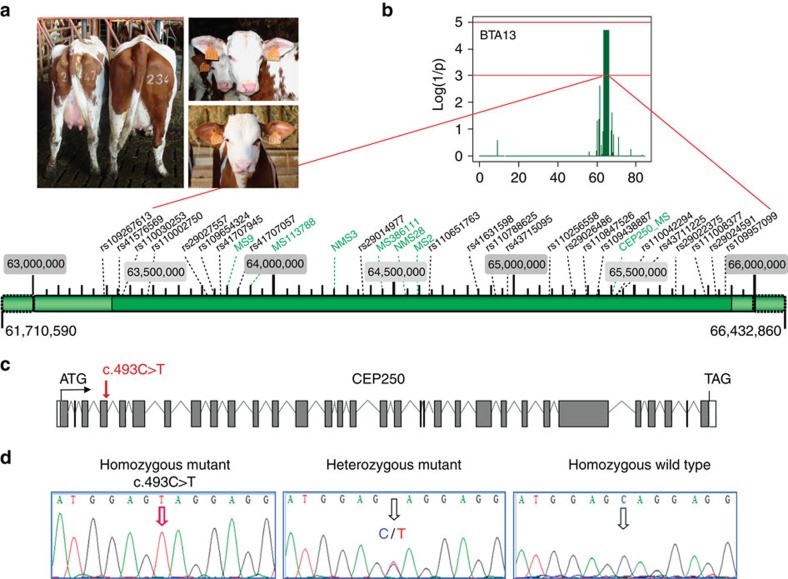
Identification of the SHGC-causing mutation in *CEP250.* (**a**) General feature of mutant cows. Left picture: backs of mutant (left) and wild-type cows (right). Right pictures: top, mutant cow with typical elongated caprine-like long and thin head with ears' depigmentation; bottom: wild-type cow. (**b**) Evidence for linkage (*y* axis) is measured as log(1/*P*), with *P* being determined by the 50,000 locus (ASSHOM) permutations (see Methods), leading to the identification of a 2.5-Mb shared region between markers BTA13 rs109267613 and rs109957099. Below, list of the analysed markers present in this region, including the microsatellites (in green) used in the initial mapping study. (**c**) The *CEP250* gene comprises 32 coding exons. The comprised 5′ and 3′ untranslated regions are depicted in white. Location of the observed mutation is indicated. (**d**) Chromatograms of the obtained sequences covering the mutation.

To refine the primary genetic location, the pedigree was extended to gather 190 affected and 200 related unaffected individuals and 7 additional microsatellites were included (Supplementary Table 1). On the basis of these results, a subset of 19 individuals identified as recombinant in the mapping region (BTA13:61,710,590-66,432,860) were genotyped on Illumina BovineSNP50 BeadChips, and homozygosity fine mapping was performed. A 2.5-Mb shared region was delineated (log (1/*P*)=4.7) between markers rs109267613 and rs109957099 (Fig. 1b).

Targeted FLX454 NGS sequencing was performed on two affected, one healthy and one carrier animals after sequence capture (BTA13: 61,710,590 to 66,432,860 mapping regions) using a Roche-NimbleGen 385-K Bovine Array (Fig. 1b). The mean sequence coverage ranged from 6 × to 11 × according to animals (Supplementary Table 2). After quality-filtering, 7,298 single-nucleotide polymorphisms (SNPs) and 3,513 InDels were identified (Supplementary Data 1). About 205 variants were predicted to have an impact on gene transcription or protein structure, on the basis of Ensembl functional annotations (Supplementary Data 2). Focusing on the critical mapping region and considering variants consistent with animal genotypes and missing in dbSNP, only eight SNPs located in five genes were retained (Table 1). None of the seven nonsynonymous mutations were assumed to be relevant on the basis of SIFT (http://sift.jcvi.org) criteria. In contrast, a C>T transition at position 65,369,074 in the *CEP250* gene coding frame leads to a premature stop codon (Fig. 1c,d). The genotype–phenotype correlation was confirmed by Taqman assay using our pedigree and 750 additional Montbéliarde sires. Furthermore, the mutation was not detected in a biodiversity panel including 316 sires from 10 French breeds.

### CEP250 mutant gene encodes N-terminally truncated C-Nap1

**Figure 2:**
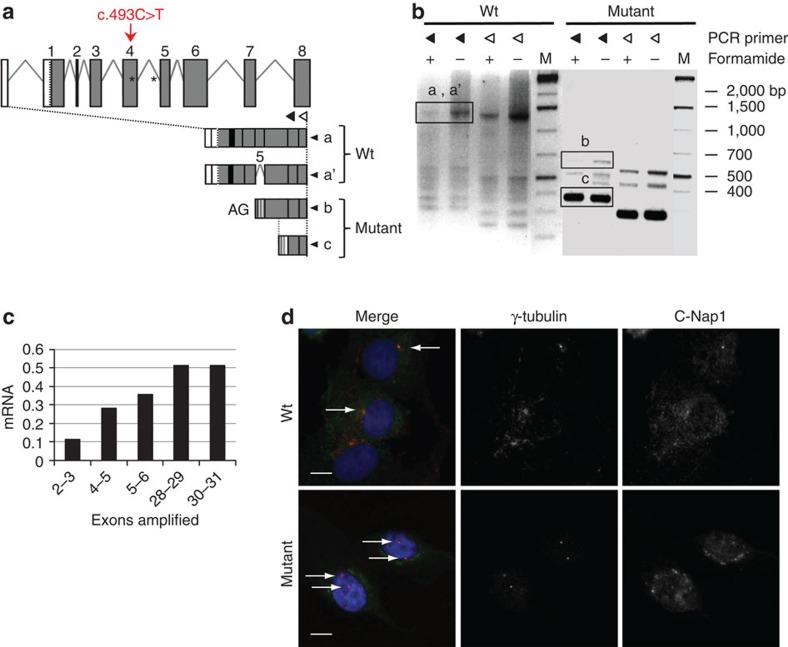
Characterization of truncated *CEP250* transcripts and C-Nap1 mislocalization in SHGC primary fibroblasts. (**a**) Structure of the 5′ region of the bovine *CEP250* gene. The 5′ untranslated region is depicted in white. Exons are numbered from the first coding exon. Two transcripts (a and a′) are detected in wild-type cells including one lacking exon 3 and retaining the last five nucleotides of intron 3 (a′). Mutation in exon 4 generates two shorter transcripts: transcript (b) starts at an AG dinucleotide likely located at the end of intron 4 and includes exon 5 (depicted as *); transcript (c) starts in exon 6 at nucleotide 656 of the open reading frame. (**b**) Agarose gel showing the 5′ RACE products amplified using RT primer GSP1 and GSP2 located in exon 9 and PCR primers GSP3 or GSP4 located in exon 8 (black and white triangles, respectively), with or without formamide. The longest products were purified and sequenced (boxes). Original gel is presented in Supplementary Fig. 6. (**c**) Relative *CEP250* mRNA levels estimated by RT–qPCR targeting the indicated exons. Values are provided relative to wild type. Mutant, *n*=3 and wild-type, *n*=2. (**d**) C-Nap1 subcellular localization in wild-type and SHGC mutant fibroblasts using C-Nap1 C-terminus labelling (R63 serum). In 92% of wild-type cells (*n*=113), centrosomes (white arrows) are labelled with an antibody against γ-tubulin (red) and with R63 (green). In 91% of mutant cells (*n*=97), only γ-tubulin labelling is detected on split centrosomes (double arrows). The anti-C-Nap1 antibody only produced a heterogeneous labelling (green colour on the merge) but no labelling of split centrosomes. Scale bar, 10 μm.

The c.493C>T mutation introduces a stop codon at amino acid 165 and is expected to be incompatible with a normal function of C-Nap1 (Fig. 1c). To unravel the effect of this mutation on *CEP250* expression, we isolated primary fibroblasts from wild-type and mutant cows and performed 5′RACE experiments and real-time quantitative reverse transcription PCR (RT–qPCR). The 5′-untranslated repeat and an exon-3-spliced variant retaining the last five nucleotides of intron 3 were characterized in wild-type fibroblasts (Fig. 2a,b). In mutant cells, only low amounts of full-length transcripts were detected (Fig. 2c), suggesting nonsense mRNA decay. In addition, shorter mRNAs starting downstream of the mutation (end of intron 4 or within exon 6) were observed (Fig. 2a–c), suggesting the occurrence of internal promoters. These transcripts contain an in-frame ATG in exons 5 and 6 that could initiate the translation of N-terminally truncated proteins (ΔN-C-Nap1) lacking at least the first 195 amino acids.

### Mutant C-Nap1 mislocalization induces centrosome splitting

**Figure 3:**
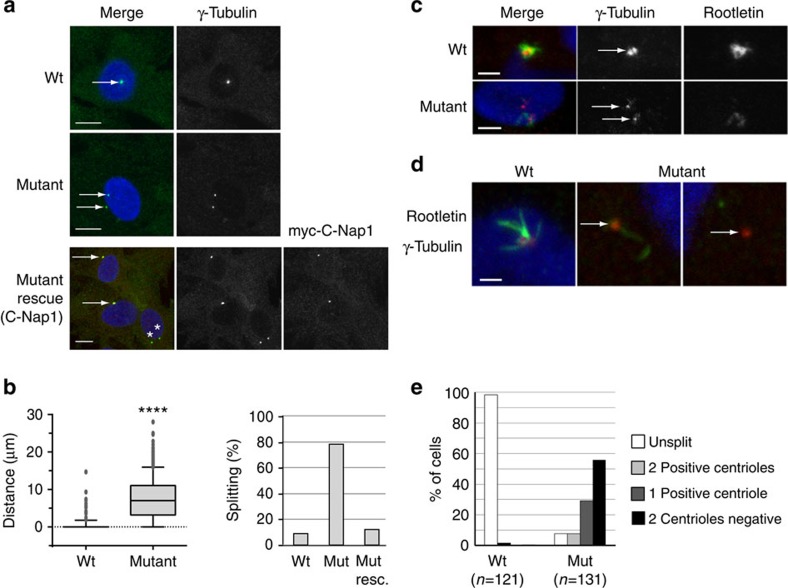
Centrosome splitting and rootletin loss in SHGC mutant cells. (**a**) Centrosomes were visualized (arrows) with gamma-tubulin immunolabelling (green) on individual fibroblasts from wild-type and SHGC mutant animals. Centrosome splitting in mutant cells was rescued by transient expression of myc-tagged human C-Nap1. Myc labelling (red) revealed unsplit centrosomes (white arrows) in 88% of the cells (*n*=42) as opposed to 21% in nontransfected cells (**, *n*=230). DNA was stained with 4,6-diamidino-2-phenylindole (DAPI). Scale bar, 10 μm. (**b**) Box and whiskers diagram: distance between two centrosomes measured on Z projections from different fields (SP5 Leica), for 344 wild-type and 686 SHGC mutant cells during exponential growth. The mean distance equals 0 for wild-type cells and 7.48 μm for SHGC mutant cells. The mean, upper and lower decile, upper and lower quartile and interquartile range of data (individual dots: outliers). Student's *t*-test following two-tailed analysis of variance (****P<0.0001). Histogram: percentage of cells with split centrosomes in 344 wild-type and 686 SHGC mutant cells, and following rescue with human C-Nap1. (**c**) Centrosome splitting and reduction in rootletin levels in the SHGC mutant kidney. Centrosomes (arrows) were visualized by immunolabelling with gamma-tubulin (red) and the intercentriolar protein linker rootletin (green). DNA was stained with DAPI. Scale bar, 10 μm. (**d**) Severe reduction of Rootletin on the centrosome of SHGC mutant fibroblasts. Wild-type cells (left panel) present a strong rootletin staining (green) in between both centrioles (gamma-tubulin in red) and extending outside the intercentriolar region. Mutant fibroblasts show either severely reduced (mid panel) or undetectable (right panel) levels of rootletin (arrows). The two latter images are from the same cell. DNA was stained with DAPI. Scale bar, 2 μm. (**e**) Quantification of rootletin staining in wild-type and mutant fibroblasts. Centrosome splitting was scored together with the presence of rootletin near the unsplit centrosome (white bar) or in any or none of the two split centrioles (grey and dark bars). Rootletin was tightly associated with the unsplit centrosome (95% of the cases) in wild-type fibroblasts as opposed to less than 10% in mutant cells. More than 50% mutant cells exhibited a split centrosome with no detectable rootletin.

The N-terminal region of C-Nap1 (amino acids 1–243) is one of the two regions that target the protein to the centrosome^6^,^9^. Thus, a mislocalization of ΔN-C-Nap1 proteins encoded by the *CEP250*-mutant gene was expected. Indeed, using the R63 serum raised against the C-terminal domain of human C-Nap1^5^, a centrosomal signal was detected by immunostaining in the majority (92%) of wild-type fibroblasts (Fig. 2d). In contrast, a weak diffuse C-Nap1 staining was observed in most mutant cells that colocalized with the centrioles in only 9% of the cases (Fig. 2d). To further unravel the mutation effect at the cellular level, we assayed centriole–centriole cohesion in proliferating and quiescent fibroblasts. In mutants, ∼80% of centrosomes were split, compared with 5–13% in wild-type cells (Fig. 3a,b and Supplementary Fig. 1). This striking defect was fully rescued by expression of low amounts of Myc-tagged human C-Nap1 in mutant fibroblast cells (Fig. 3a,b). In addition, centrosome splitting occurred independently of the cell cycle phase and was also observed on quiescence in mutant cells (Supplementary Fig. 1). Centrosome splitting was also observed in kidney sections from homozygous mutant cattle (Fig. 3c). Surprisingly, centrosomes remained cohesive in 10–20% mutant cells, suggesting some heterogeneity at the cellular level. Three hypotheses may be drawn to explain this feature. First, low amounts of functional C-Nap1 may be synthesized by stop codon read-through of the mutant mRNA. Second, alternative wild-type mRNAs may be produced by alternative splicing or from alternative promoters. Finally, mechanisms independent of C-Nap1 and the intercentriolar tether may also contribute in maintaining centrosome cohesion, similar to microtubule and microfilament-dependent mechanisms^10^. In conclusion, the c.493C>T mutation producing a shorter C-Nap1 protein leads to an almost complete disruption of its localization and loss of centriole–centriole cohesion, a phenotype more pronounced than previously obtained with antibody injection or RNA interference experiments^6^,^25^.

### Mutant C-Nap1 impacts cilliary rootlet organization

**Figure 4:**
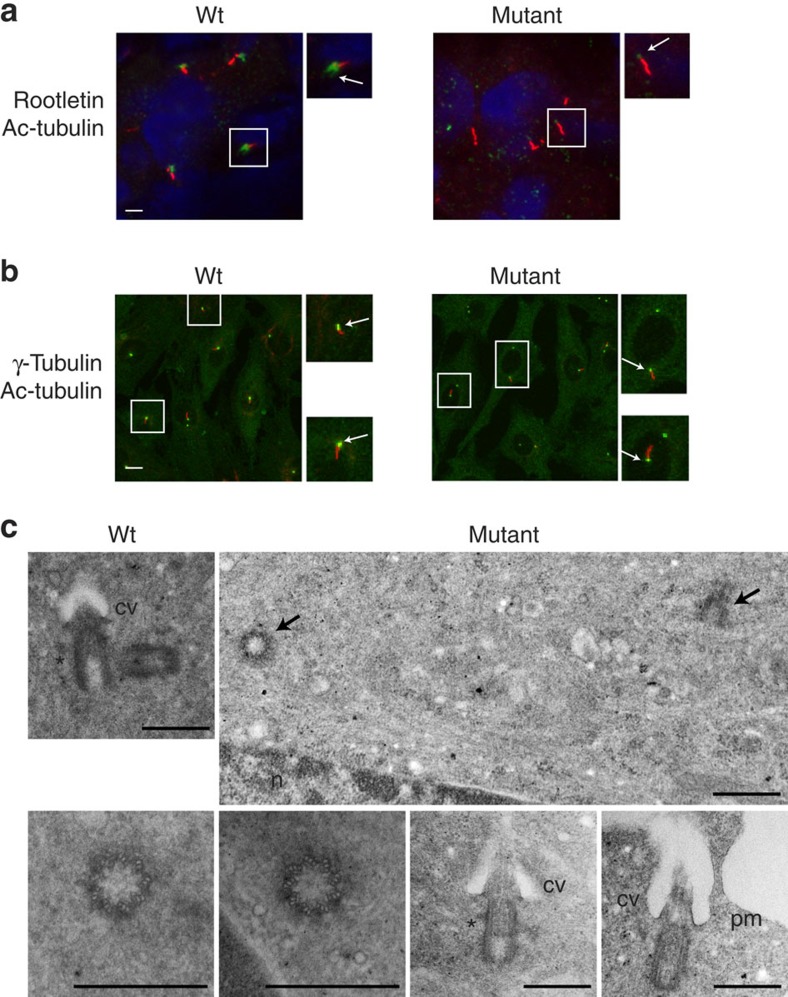
Ciliogenesis and ultrastructure of mutant centrioles observed using transmission electron microscopy. (**a**) Ciliogenesis *in vivo* in adult kidney cells. Cilia were observed on kidney sections from wild-type and mutant cattle using acetylated tubulin to label the axoneme (in red) and rootletin (in green, arrow) to stain the cilium base. Enlargements show a cilium with strongly reduced rootletin staining in the mutant context. Distribution of cilia in SHGC mutant tissue was similar to the wild type, despite the very severe reduction of rootletin labelling (white arrow). Scale bar, 10 μm. (**b**) Ciliogenesis in fibroblast cells with split centrosomes. Cilia presence and length were analysed after labelling the axoneme with acetylated tubulin (in red) and the basal body with gamma-tubulin antibodies (in green, arrow). Enlargements of cells are shown to highlight the cilia. Mutant cells have the ability to grow and maintain cilia as efficiently as wild-type cells (see Supplementary Fig. 3 for statistics). Scale bar, 10 μm. (**c**) Normal centriole ultrastructure and cilium assembly in SHGC mutant fibroblasts. Wild-type centrioles were found as pairs in 50% of the sections analysed (*n*=20/42), while mutant centrioles were found isolated in 93% of the sections examined (*n*=91/98) or distantly located (black arrows). Transverse sections through the basal body shows the normal 9 microtubule triplets arrangement in the mutant centrioles and longitudinal sections allows the detection of subdistal appendages (*) and distal appendages that allow ciliary vesicle docking (cv), and subsequent fusion with the plasma membrane (pm). n, nucleus. Scale bar, 500 nm.

To determine whether C-Nap1 mutation affects the localization of its interacting partners, we analysed the linker protein rootletin and Cep135, the latter being the docking protein for C-Nap1 on the centriole. Figure 3c–e shows that rootletin localization near the centrioles was strongly reduced in mutant kidney cells, as well as in primary fibroblasts. In addition, we observed a disorganization of the ciliary rootlet in ciliated cells from the mutant kidney (Figs 3c and 4a), a characteristic feature of rootletin-deficient cells^26^. These results are highly consistent with rootletin being dependent on the presence of C-Nap1 as a docking site in the centriole. In contrast, Cep135 localization on the centriole appears independent of its interaction with C-Nap1 (Supplementary Fig. 1b).

**Figure 5:**
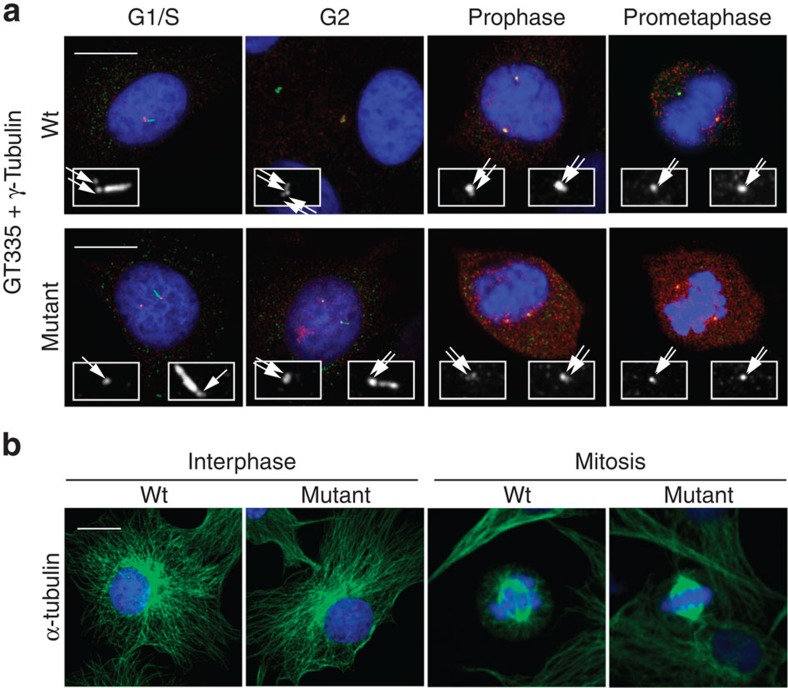
Bipolar mitotic spindle formation with paired centrioles in SHGC mutant cells. Images show examples of microtubule, centrosome and centriole staining in wild-type and SHGC mutant primary fibroblasts at different stages of the cell cycle. (**a**) Newly formed centrioles remain associated with parental centrioles through the cell cycle in C-Nap1-deficient cells. Fibroblasts were synchronized in G1/S with thymidine and released for 8 h to observe cells in G2 or in various mitotic phases such as prophase and prometaphase. Centrioles and cilia are labelled with antiglutamylated tubulin monoclonal antibody GT335 (green), the centrosome with anti-gamma-tubulin serum (red), nuclei are stained with DAPI (blue). Insets are enlargements of GT335 labelling to highlight the number of centrioles (two centrioles in G1/S and four centrioles in G2 and mitosis). Note the presence of cilia in some G1/S and G2 cells. White arrows point each centriole. Merged images are stacks of four sections of 0.2 μm. Scale bar, 10 μm. (**b**) C-Nap1-deficient cells do not exhibit major defects in microtubule organization in interphase or mitosis. Asynchronously growing fibroblasts were immunolabelled for alpha-tubulin in green and DNA was stained with DAPI (blue). *N*=50 mitotic mutant cells were examined to assess spindle bipolarity.

As the SHGC bovine phenotype shows features of MCPH and Seckel syndrome, we examined centrioles in proliferating primary fibroblasts. First, electron microscopy was performed to analyse centriole morphology (Fig. 4c), showing that SHGC fibroblast centrioles were indistinguishable from wild-type centrioles. From a series of 98 mutant and 42 wild-type cells analysed, we could observe that centrioles exhibited a normal diameter, length and structure with the conventional nine triplets of microtubules. Centrosome splitting was also confirmed at this level of observation (Fig. 4c). Second, we used immunofluorescent labelling in synchronized cells to follow centriole duplication during the cell cycle. Figure 5a shows that mutant cells arrested in G1/S before the onset of centriole duplication exhibit two separated centrioles, while in G2 and mitosis four centrioles organized in two pairs (a mother and a daughter) are seen. This observation is consistent with previous studies, suggesting that C-Nap1 is not implicated in the tether between mother and daughter centrioles during the duplication process that starts in the S phase. Therefore, neither centriole duplication nor association of mother and nascent daughter centrioles were affected in SHGC-proliferating mutant cells. SHGC fibroblasts did not exhibit obvious defects in ploidy, cell cycle entry and proliferation (Supplementary Fig. 2). Consistently, mutant cells were able to assemble a bipolar mitotic spindle and to activate the spindle assembly checkpoint when active centrosome separation, normally occurring in prophase, was inhibited by addition of monastrol, an inhibitor of the kinesin-related protein Eg5 (ref. 27; Supplementary Fig. 3). No dysmorphic nuclei were observed and the microtubule cytoskeleton appeared organized (Fig. 5b), although it would be premature to exclude subtle effects on microtubule dynamics. Therefore, SHGC fibroblasts harbouring centrosome splitting are not prevented from undergoing cell division and proliferation at normal rate, in agreement with previous findings^6^. Although we failed to observe marked defects on mitosis in fibroblasts harbouring mutant C-Nap1, we cannot exclude that impaired centrosome cohesion may produce more pronounced effects on cell cycle progression in other cell types. This view is supported by recent studies demonstrating that the timing of centrosome separation affects the kinetics of mitotic progression and the rate of chromosome missegregation to different degrees in different cell lines^28^,^29^. Thus, further studies will be required to fully understand the complete spectrum of phenotypes produced by C-Nap1 mutations in different genetic backgrounds, cell types and physiological states.

Since the role of C-Nap1-mediated centrosome cohesion in ciliogenesis is unclear^25^,^30^, ciliogenesis was analysed in SHGC fibroblasts either at confluence without serum or during exponential growth. In both cases, ciliogenesis occurred efficiently in mutant fibroblasts. Percentage of ciliated cells in mutant versus wild type was either unchanged or higher, despite a great heterogeneity in affected animals (Fig. 4b and Supplementary Fig. 4). Consistent with these observations, ultrastructural analysis of centrioles revealed that mother centriole had appendages that allowed ciliary vesicle docking and axoneme assembly (Fig. 4c). Finally, analysis of cilia in kidneys from mutant cows confirmed their presence and normal length in that organ, even if rootletin was either missing or severely reduced at the cilium base (Fig. 4a). Cilia loss is thus not likely to account for the SHGC phenotype.

### SHGC mutation alters fibroblast directional migration

**Figure 6:**
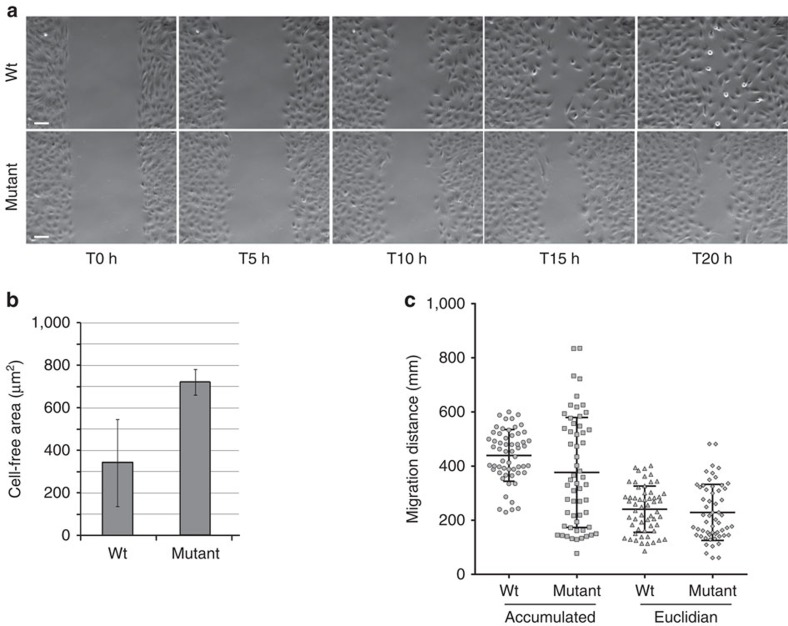
Altered migration behaviour in SHGC mutant fibroblasts. (**a**) Phase contrast images from time-lapse video-microscopy showing the migration capacity of cells in a wound-healing assay. Wild-type and SHGC mutant confluent quiescent cells migrated into the cell free gap of 500 μm during 20 h. Scale bar, 100 μm. (**b**) The cell-free area was measured at 20 h. Data show the mean and standard deviation from four independent experiments with one animal of each genotype. A Student's *t*-test following two-tailed analysis of variance using the Prism software was run to calculate *P* values (**P=0.008). (**c**) The migration tracks of wild-type and SHGC mutant cells were plotted in ImageJ (manual tracking plugin) and data analysed after normalizing each starting point to *x*=0 and *y*=0 (Ibidi software). The accumulated and Euclidean distance covered by individual cells, as well as the mean, upper and lower decile of the directionality distribution from four individual experiments of two wild-type and two mutant SHGC cell cultures are shown. Wild-type cells, *n*=73. SHGC mutant cells, *n*=52. A Student's *t*-test following two-tailed analysis of variance using the Prism software was run to calculate *P* values (****P<0.0001). The greater the directionality, the more linear the motion in a given direction is.

To determine whether C-Nap1-mediated centrosome cohesion is required for directional cell migration, we examined the cell migration ability of SHGC fibroblasts using a wound closure assay (Fig. 6a,b). Results show that SHGC cells are less efficient than wild-type cells in closing the wound, mainly because of reduced directionality of movement (Fig. 6c and Supplementary Fig. 5). Reduced directionality of mutant cells was also observed in a real-time directional migration assay using 10% serum as an attractant (Supplementary Fig. 5). How exactly the loss of C-Nap1 function affects cell directionality will require additional studies. Cell polarization at both the plasma membrane and the Golgi is an important determinant of directional migration^31^,^32^. As Golgi polarization depends on centrosome position, any alteration in centrosome structure could impact on Golgi behaviour, polarization and migration^33^. A migration defect in SHGC cells might account for the pigmentation impairment and the facial dysmorphism. Similarly, migration defect of neural crest cells during early development may indirectly explain hind limb muscular hypoplasia as it plays a critical role in sustainable myogenesis^34^. This hypothesis remains to be fully substantiated *in vivo*.

### CEP250 gene mutation in humans

On the basis of features reminiscent of Seckel syndrome, we searched for mutations in 13 human Seckel patients with unknown underlying genetic mutations. Our exome analysis failed to identify any *CEP250* mutation in this small set of patients. Recently, a homozygous nonsense mutation in the C-terminal part of the C-Nap1 protein (R1155*) in combination with a C2orf71 nonsense mutation have been associated with retinitis pigmentosa and sensorineural hearing loss in humans^35^. Since SHGC cattle did not suffer from apparent ocular or hearing defects and no mutation could be identified in the Montbéliarde C2orf71 gene, the nonsense mutation in C2orf71 may be the pathogenic allele in a mutated C-Nap1 genetic background in humans, in good agreement with the observed additive effect of this mutation. Alternatively, locations of the mutations in the *CEP250* gene may explain the different phenotypic outcomes, in agreement with structure/function analyses of the C-Nap1 protein^6^,^36^.

In conclusion, we have shown that the mutation c.493C>T in the *CEP250* gene coding for the C-Nap1 protein causes autosomal-recessive SHGC in cattle. In contrast to mutations of CEP135 (ref. 37), CEP152 (ref. 17) or STIL^21^ that affect centrosome number, this mutation solely affects centrosome cohesion. Thus, C-Nap1-mediated centrosome cohesion is not required for centriole duplication, cell division and cilia assembly in fibroblasts, but seems to affect the directionality of cell movement *in vitro*. However, the absence of any strong phenotype in fibroblasts does not exclude that more subtle defects have been missed, that may affect more severely other cell types. It is also possible that N-terminal truncation of C-Nap1 may confer some functionality to the protein and a detailed analysis of the role of splice variant isoforms of C-Nap1 might be rewarding. The clinical features (facial dysmorphism, low birth weight, short stature and pigmentation anomaly) associated with the *CEP250* c.493C>T mutation make SHGC the first described Seckel-like syndrome in cattle and extend the range of loci potentially involved in similar pathologies in humans.

## Methods

### Ethics statement

Experiments reported in this work comply with the Institut National de la Recherche Agronomique ethical guidelines. Montbéliarde blood samples were collected by trained and licensed technicians during routine blood sampling for paternity testing, annual prophylaxis or genomic selection purpose. Montbéliarde sperm was obtained from semen straws collected by approved commercial artificial insemination stations, as part of their regular semen collection process. Tissues were sampled on 3- to 5-year-old cows, either at slaughterhouse (controls) or at an Institut National de la Recherche Agronomique experimental station (SHGC) under experimental approval number C16-157-001. All samples and data were obtained with the permission of breeders or breeding organizations.

### Sample selection and DNA extraction

The diagnosis of SHGC in Montbéliarde cattle was based on established, previously published criteria^22^. A total of 190 affected and 200 related unaffected animals were collected. In addition, 750 Montbéliarde sires were collected, as well as 316 animals from 10 French breeds (6 Bazadaise, 25 Bretonne-Pie-Noire, 48 Charolaise, 13 Gasconne, 36 Limousine, 31 Maine-Anjou, 44 Maraichine, 50 Normande, 26 Parthenaise and 37 Holstein). EDTA blood samples were collected by licensed agricultural technicians. Sperm was obtained from semen straws provided by Umotest and Jura-Bétail breeding companies. DNA was extracted from blood samples with the Genisol Maxi-Prep kit (AbGene, UK) or from semen with a phenol/chloroform extraction (Miller, SA). DNA quality was assessed via 2% agarose gel electrophoresis. DNA concentration was measured using the NanoDrop ND-1000 spectrophotometer.

### Genotyping and homozygosity mapping

Samples were genotyped using the Illumina BovineSNP50 BeadChip as recommended by the manufacturer. Data were analysed with the GenomeStudio software and individuals with a call rate below 95% were discarded from further analyses. Homozygosity mapping was performed using ASSHOM^38^. Briefly, this approach consists of scanning the genome for regions of homozygosity shared by affected animals. To account for the population allele frequencies, data from the whole genotyped Montbéliarde reference population was used and frequencies were adjusted for close relationships^39^. The ASSHOM statistics then allows summarizing this information over all affected animals (via a harmonic mean) in such a way that the longer and rarer the homozygous haplotype, the higher the score. The whole-genome significance of the ASSHOM statistics was assessed with *n*=50,000 random permutations of the haplotypes^38^.

### High-throughput sequencing of SHGC genomic interval

The 5-Mb SHGC mapping region was selected for enrichment using a Roche-NimbleGen custom Sequence Capture 385-K Bovine Array, on the basis of the Bos Tau4 assembly and Human/Bovine comparative maps. The targeted region was tiled to avoid capturing repetitive DNA fragments. About 10 μg of genomic DNA was sonicated to a 300- to 500-bp size range, purified (Agencourt AMPure XP system) and quality-controlled on Bioanalyzer 2100 (Agilent). Fragments were then ligated to universal gSel3 and gSel4 adapters (Roche NimbleGen) with T4 DNA Ligase. Small fragments (<100 bp) were removed with the use of Agencourt AMPure Beads. The resulting library was hybridized to the custom 385-K array, and captured DNA was eluted and amplified using ligation-mediated PCR according to the manufacturer's instructions.

### Next-generation sequencing and data analysis

Sequencing was performed at the Get-PlaGe facility (http://genomique.genotoul.fr/) on a Roche 454/FLX Genome Sequencer platform using the GS FLX Titanium chemistry. NimbleGen primer sequences were removed. Reads were filtered according to their quality, sequence length (100–500 bp), duplication and complexity and mapped on the UMD3.1 reference genome using the BWA software. After filtering for alignment quality (MAQ score⩾10), variants were predicted using SAMtools (mpileup). InDels were filtered for homopolymers generated because of a known artefact from Roche 454 technology. The analysis relied on one healthy animal expected to be homozygous for the reference allele, one carrier animal expected to be heterozygous for the variant and two affected animals expected to be homozygous for the alternative allele. Thus, besides filtering the vcf file for SNP quality (⩾10 and coverage ⩾4), SNPs could also be selected on the basis of the phenotype–genotype correlation.

### Validation of the mutation using the Taqman assay

In order to validate the putative causal SNP, a Taqman assay was performed according to the manufacturer's recommendations (Applied Biosystems). Specific SNP probes (SHGC_V CTTACCTCCTGCTCCATC (VIC) and SHGC_F TCTTACCTCCTACTCCATC (FAM)) and primers (SHGC_Fd CCGGGATGAGCTAATGAGGAA and SHGC_Rv GGCCGTGCCCAACCT ) were obtained from Applied Biosystems Assay-by-Design Service for SNP genotyping. Allelic discrimination was performed with the ABI PRISM 7900 HT using the Sequence Detection Software (SDSv2.3, Applied Biosystems). PCR reactions were performed in a 6-μl final volume, using the following cycling protocol: 94 °C—10 min and (94 °C—15 s, 60 °C—1 min) for 50 cycles.

### Primary fibroblast isolation, culture and synchronization

Fibroblasts were isolated from ear punches of four SHGC homozygous mutant and four wild-type Montbéliarde cows. Cells were seeded in T75 flasks with 30% Amniomax II (Gibco, Life Technologies) and then passaged twice a week with 1/3 dilution in minimum essential medium (MEM, Gibco) containing 15% fetal calf serum with and antibiotic–antimycotic (Gibco). Cells at passages 4–6 were used for the experiments. Cells were either grown asynchronously or density-arrested. Cells were synchronized in G1/S with 2 mM thymidine block for 24 h and then released for 8 h to observe cells in G2 and mitosis. Cells were also released in the presence of 0.1 mM monastrol (Tocris Biosciences) to arrest them in metaphase with a monopolar spindle.

### 5′ RACE-PCR

Total RNA was extracted from fibroblast cultures using Trizol reagent (Invitrogen) and the RNeasy mini kit (Qiagen) with DNase I treatment. About 5 μg RNAs were reverse-transcribed using GSP1 primer and SuperScript II kit (Invitrogen), according to the manufacturer's instructions. First-strand cDNA was treated by RNase H, purified on the GlassMax-DNA isolation Spin cartridge system (Gibco-BRL) and poly-C tailed using dCTP and terminal transferase (Gibco-BRL). Thirty-five PCR cycles were performed using AAP anchor primer (Gibco-BRL) and C-Nap1-specific primers GSP2. An aliquot of these PCR products was used in a second PCR amplification run, using primers AUAP and GSP3 or GSP4. PCR primers are detailed in Supplementary Table 1. PCR reactions were electrophoresed on a 1% agarose gel. Amplified DNA fragments were cloned into pGEM-T (Promega) and sequenced.

### Quantitative RT–PCR

Reverse transcription was performed on 500 ng of total RNA using the Vilo kit (Invitrogen). Quantification was performed on triplicates using the ABsolute Blue QPCR SYBR Green ROX Mix (Thermo Scientific). Primers (Supplementary Table 1) were designed using the Primer3 software (http://primer3.ut.ee) on separate exons to produce 100-bp amplicons avoiding DNA amplification. The GAPDH gene was used for normalization based on its observed expression stability in fibroblast cells.

### Immunolabelling of centrosome proteins and cilia

Cells grown on gelatin-coated coverslips were fixed in methanol for 7 min at −20 °C, rinsed in PBS and incubated overnight with monoclonal antibodies directed against gamma-tubulin (Sigma GTU88 at 1:500), acetylated tubulin (Sigma T6793 at 1:500), R145/anti-rootletin rabbit serum (1:500; gift from Dr E. Nigg), anti-Clam Cep135 polyclonal serum (gift from Dr R. Kuriyama, at 1:10,000) or Myc epitope (9B11, Cell Signalling, 2,276 at 1:200) in PBS containing 0.1% Tween-20 and 10% fetal calf serum. Cryostat tissue sections (kidney, 5 μm) were processed similarly. Secondary antibodies were from Molecular Probes (1:400) and nuclei were stained with 4,6-diamidino-2-phenylindole. Coverslips were mounted in Vectashield (Vector) before image acquisition with a SP5 Leica Confocal microscope (× 63 objective). Z stacks were taken every 0.25 μm from different fields. Distance between gamma-tubulin spots and cilia length was measured using the ImageJ Software on Z projections. Affected animals (*n*=3) were compared with controls and measurements were performed on 100–150 cells for each animal. Statistics were calculated with the R Software. For quantification of the immunolabelling of Cep135 at centrioles, the ratio between the mean intensity of gamma-tubulin staining and the mean intensity of Cep135 staining was calculated using three-dimensional (3D) Image Object Counter in the Fijisoftware. Statistical analysis was performed using an unpaired *t*-test with Welch's correction in the Prism 6 software. For rescue experiments, mutant cells grown to 50% confluency in 12-well plate were transfected with 1 and 0.3 μg of human Myc-tagged C-Nap1 expression vector (gift from Dr E. Nigg)^6^ and 3 μl of X-TremeGENE HP DNA transfection reagent (Roche). Two days after transfection, human C-Nap1-expressing cells were visualized owing to the Myc staining. Rescue of centriole splitting was analysed with the lowest concentration, in which no aggregates of human C-Nap1 were formed.

### Immunolabelling of C-Nap1, centrioles and microtubules

Cells were fixed for 3 min in cold methanol or 4% paraformaldehyde, stained, mounted and observed as described above. Primary antibodies were mouse monoclonal anti-acetylated tubulin (Sigma T6793 at 1:500), R63/anti-C-Nap1 rabbit serum (a gift from Dr E. Nigg, 1:500)^5^, rabbit monoclonal antibodies against poly-glutamylated tubulin (GT335, a gift from Dr B. Eddé, 1:5,000)^40^ and α-tubulin (DM1A Sigma-Aldrich, 1:500). Images were acquired on a Leica DMI 6000B microscope system (Leica Microsystems) equipped with a × 63 objective and a Quantem 512SC CCD camera. Images were captured in Metamorph and analysed in the ImageJ software. Alternatively, cells labelled with R63 were acquired on a Zeiss Axio Observer.Z1 microscope equipped with a × 40 objective and a CoolSNAP HQ2 camera (Photometrics). Images were captured in AxioVision (Zeiss).

### Cell migration

SHGC and wild-type fibroblasts were seeded on Ibidi Culture Inserts (Ibidi GmbH). Ibidi inserts were removed and closure of the resulting wound was monitored over the next 24 h. Images of the wound were captured using the Leica DMI 6000B microscope system equipped with a × 10 objective and Nomarski optics. Images were captured in Metamorph and analysed in the ImageJ software. Cell-free surface was calculated with the ImageJ software, by manually tracing the area over time.

Cell migration tracks were analysed with ImageJ using the ‘manual tracking plugin' and the ‘chemotaxis and migration tool plugin' from Ibidi. In each experiment, 12–20 individual cells were tracked per sample. Migration efficiency was quantified by calculating the Euclidean (linear distance between the start and the end points) and accumulated distances (total distance) of each individual cell and the directionality of migrating cells. Directionality denotes the ratio of the linear distance from the starting point to the end point and the total distance traversed by the cell (Euclidian versus accumulated distance). Alternatively, directional migration was analysed in real time using the xCELLigence platform (Roche Applied Science, Germany) and the CIM-16 plate. Cells were seeded in the upper chamber in a serum-free medium and attracted to the bottom chamber with serum-containing medium. Impedance was measured every 10 min over 10 h.

### Cell proliferation and cell cycle analysis

Asynchronous cells were pulse-labelled for 15 min with EdU (5-ethynyl-2′-deoxyuridine) and then fixed and stained to visualize cells in the S phase as described by the manufacturer (Invitrogen). Cells were also labelled with MPM-2 monoclonal antibody (1:500, Abcam) to visualize mitotic cells. DNA was stained with 10 μg ml^−1^ propidium iodide in the presence of 0.1 mg ml^−1^ RNAse A (Sigma-Aldrich). Fluorescence was acquired using a MACS-Quant flow cytometer (Miltenyi), and data were processed in MACS-Quantify (Miltenyi) and Venturi-One (Applied Cytometry Systems) softwares. Cell ploidy was inferred from DNA contents in confluent quiescent cells. The growth curve was established from triplicate cultures initiated at the density of 2 × 10^3^ cells per cm^2^ and counted every 24 h over 5 days using a MACS-Quant flow cytometer (Miltenyi).

### Electron microscopy

For transmission electron microscopy, the cells were washed in 0.1 M phosphate buffer, pH 7.4 and fixed in 2% glutaraldehyde for 1 h at room temperature. The samples were first washed in 0.1 M phosphate buffer and then in bidistilled water and finally postfixed in 1% osmium-bidistilled water for 1 h at room temperature. After washes in bidistilled water, the samples were dehydrated in increasing concentrations of ethanol, infiltrated in 1:1 ethanol:epon resin for 1 h and finally 100% epon resin for 48 h at 60 °C for polymerization. Seventy-nm-thick sections were cut with an ultracut UCT microtome (LEICA) and picked up on copper rhodium-coated grids. Grids were stained for 2 min in Uranyless (DELTA Microscopies) for 5 min in 0.2% lead citrate. Grids were analysed on an electron microscope (EM 912 OMEGA, ZEISS) at 80 kV, and images were captured with digital camera (Side-Mounted TEM CCD, Veleta 2kx2k). The software used is iTEM.

## Author contributions

S.F., A.E., D.B. and L.S. conceived and managed the project. Molecular genetics was performed by S.F., M.G., M.B. and A.D. Haplotypes were generated by S.Fr. and D.B. S.R. and L.S. carried out the bioinformatics analyses. SNP genotyping and mutation scanning were carried out by A.V. M.M., S.F. and S.H. performed protein experiments and provided several antibodies. V.C.-D. recruited patients affected by Seckel syndrome and J.E.H. realized the *CEP250* exome sequencing. E.A.N. provided R63 antibody and the C-Nap1 full-length vector for the rescue. J.S.-T. conceived and managed the cell biology part of the project. F.B.-G. conceived and performed cell cycle and centriole analyses. C.V. analysed centrosome splitting, ciliary length, conceived and performed rescue experiments. S.S.-M. participated in the conception and analysis of the cell biology experiments. G.T. performed the electron microscopy. A.K. and F.B.-G. conceived and analysed cell migration and wound-healing experiments. R.G. and N.C. performed the detailed necropsy, including X-Ray imaging as well as MRI. Experimental animals were managed by S.B. L.M. was involved in the initial discovery of the disease and in sample collection. All authors contributed to writing the manuscript.

## Additional information

**Accession codes**: All sff data files have been deposited in NCBI Sequence Read Archive (SRA) under the sample accession codes SRS845153, SRS845154, SRS845156 and SRS845157.

**How to cite this article:** Floriot, S. *et al*. C-Nap1 mutation affects centriole cohesion and is associated with a Seckel-like syndrome in cattle. *Nat. Commun.* 6:6894 doi: 10.1038/ncomms7894 (2015).

## Supplementary Material

Supplementary Figures and Tables Supplementary Figures 1-6 and Supplementary Tables 1-2

Supplementary Data 1 List of variants after quality filtration, associated with their Ensembl (VEP) functional annotations. Columns provide all relevant information, such as chromosome number (CHROM), Position (POS in bp), Reference and alternative allele (REF and ALT), variant quality (QUAL) and type (TYPE), global read depth at the position (DP_all) as well as genotype (GT), read depth (DP) and genotype quality (GQ) for each sample. Ensembl gene name (Gene) and predicted impact of the variant (Consequence) are also shown.

Supplementary Data 2 List of 205 variants predicted to impact gene transcription or protein structure, based on Ensembl functional annotations. Columns provide all relevant information, such as chromosome number (CHROM), Position (POS in bp), Reference and alternative allele (REF and ALT), variant quality (QUAL) and type (TYPE), global read depth at the position (DP_all) as well as genotype (GT), read depth (DP) and genotype quality (GQ) for each sample. Ensembl gene name (Gene) and predicted impact of the variant (Consequence) are also shown.

## Figures and Tables

**Table 1:** Candidate SNP in the SHGC critical mapping region of bovine chromosome 13.

**POS**	**REF**	**ALT**	**Gene**	**Description**	**Consequence**	**Amino-acid substitution**
63329680	T	C	ENSBTAG00000031361	Uncharacterized protein	Nonsynonymous coding	Tyr/His
63346834	A	G	ENSBTAG00000031354	Uncharacterized protein	Nonsynonymous coding	Ile/Val
63346948	G	A	ENSBTAG00000031354	Uncharacterized protein	Nonsynonymous coding	Val/Ile
63351273	C	G	ENSBTAG00000031354	Uncharacterized protein	Nonsynonymous coding	Leu/Val
63398648	A	C	ENSBTAG00000018535	CDK5 regulatory subunit-associated protein 1	Nonsynonymous coding	Leu/Arg
63758326	G	T	ENSBTAG00000039313	ZNF341 protein	Nonsynonymous coding	Ala/Ser
63772681	G	A	ENSBTAG00000039313	ZNF341 protein	Nonsynonymous coding	Gly/Ser
65369074	C	T	ENSBTAG00000006021	centrosomal protein 250kDa	Stop gained	Gln/Ter

SHGC, Caprine-like Generalized Hypoplasia Syndrome; SNP, single-nucleotide polymorphism.

Position (POS) in base pairs of candidate nucleotide polymorphisms (reference allele REF and alternative allele ALT) are indicated, as well as the deduced amino acid resulting substitutions.
