# Supplementary material for: C-Nap1 mutation affects centriole cohesion and is associated with a Seckel-like syndrome in cattle
Source: Nat Commun. 2015 Apr 23;6:6894. doi: 10.1038/ncomms7894 (PMC4423223; doi:10.1038/ncomms7894)
Supplement: Supplementary Figures and Tables — Supplementary Figures 1-6 and Supplementary Tables 1-2 [file ncomms7894-s1.pdf]

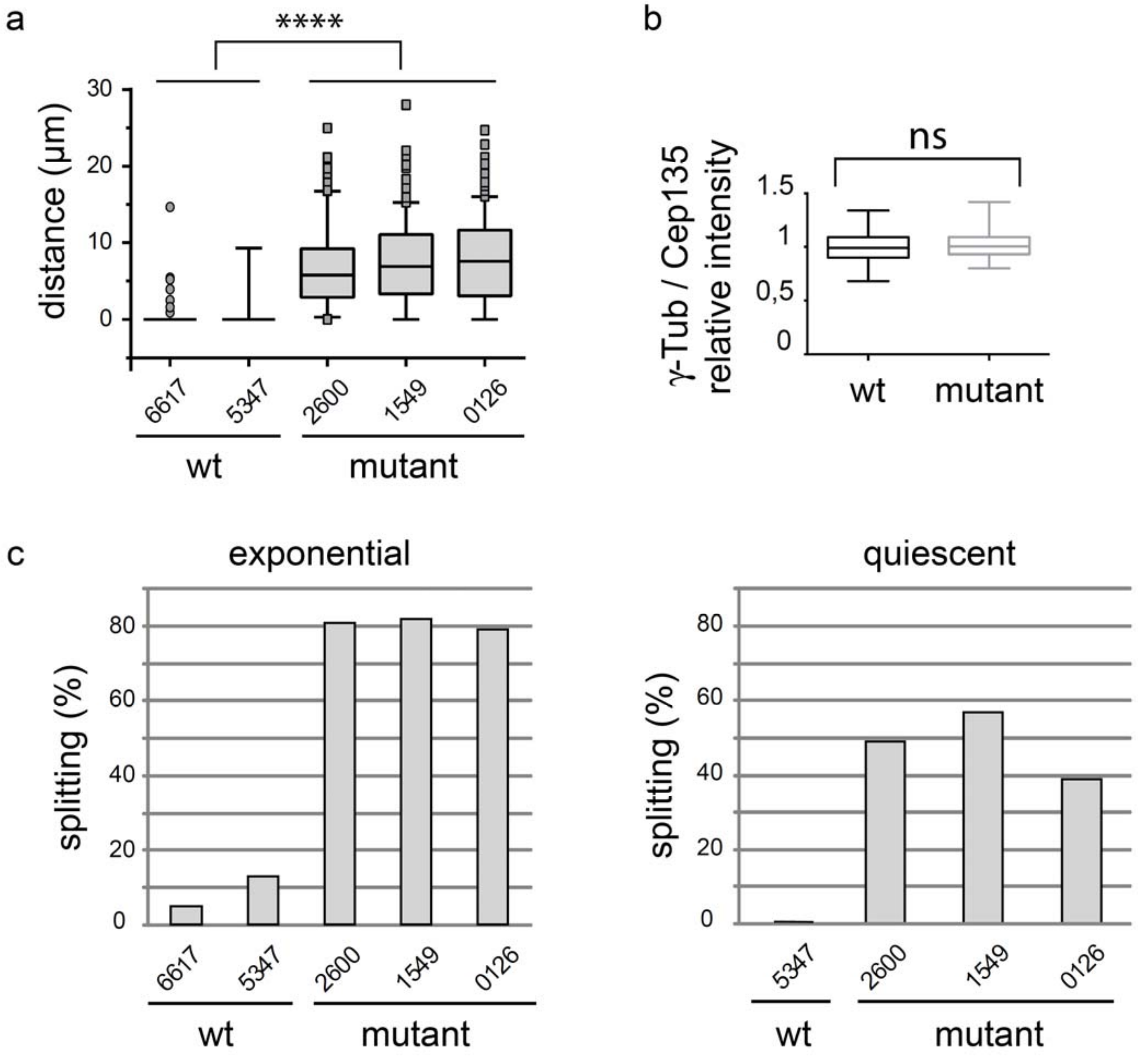

Supplementary Figure 1: Centrosome splitting quantification for individual mutants

a. Box and whiskers diagram representing the distance between the two centrosomes (gamma-tubulin positive structures) for each cell on Z-stack projections from different fields acquired with a SP5 Leica Confocal (ImageJ software) for wild type cells (n= 190 for 6617 and n= 154 for 5347) and for SHGC mutant animals (n= 228 for 2600; n= 210 for 1549; n= 248 for 0126) in exponential growth conditions. Data show mean, upper and lower quartile and inter-quartile range of data, outliers from 10-90 percentile values appear as individual dots. A Student's t-test using Prism software following two-tailed analysis of variance was run to calculate P values (****; P< 0.0001).

b. Box and whiskers diagram representing quantification of the immunolabelling of Cep135 at centrioles in wild type and SHGC mutant fibroblasts. The ratio between the mean intensity of gamma-tubulin staining and the mean intensity of Cep135 staining was calculated using 3D Image Object Counter in Fiji software. Statistical analysis was performed using an unpaired t-test with Welch's correction in Prism 6 software. ns: no significant difference.

c. Lower panels show the percentage of cells with split centrosomes during exponential growth (left) or during quiescence at confluence with serum deprivation (right). A cell was counted as having a split centrosome if the distance between the 2 gamma-tubulin dots was greater than 2 µm. For the exponential growth condition, a total of n=344 wild type cells (n=190 for animal 6617; n= 154 for animal 5347) and n=686 mutant cells were analyzed (n=228 for animal 2600; n=210 for animal 1549 and n=248 cells for animal 0126). For the quiescent phase analysis, a total of n=123 wild type cells and n= 402 mutant cells were analyzed (n=94 for animal 2600; n=151 for animal 1549 and n=157 for animal 0126).

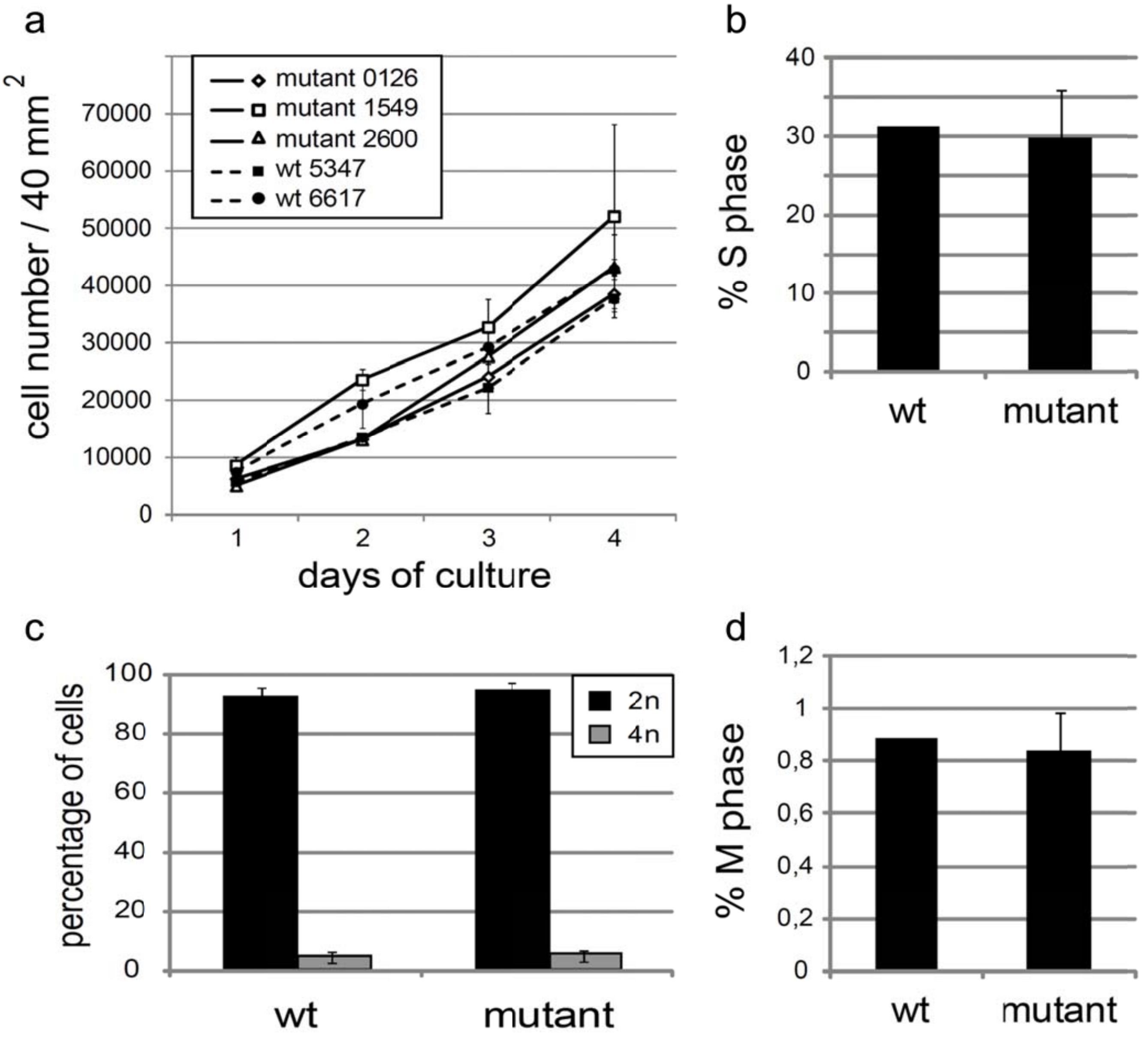

Supplementary Figure 2: SHGC mutant fibroblasts show no defect in proliferation and DNA content

a. Growth curves showing that wild type and mutant cells have a similar doubling time of about 24 hours. The graph represents the mean of 3 experiments done in triplicate.

b. Histogram showing the distribution of cells in S phase (following EdU incorporation and detection).

c. Histogram showing the ploidy of cells as measured by DNA content analysis in quiescent cells.

d. Histogram showing the distribution of cells in M phase (positive for the mitotic phospho-epitope MPM2).

Experiments were performed in triplicate in cells from at least two different animals of each genotype. Error bars represent s.d.

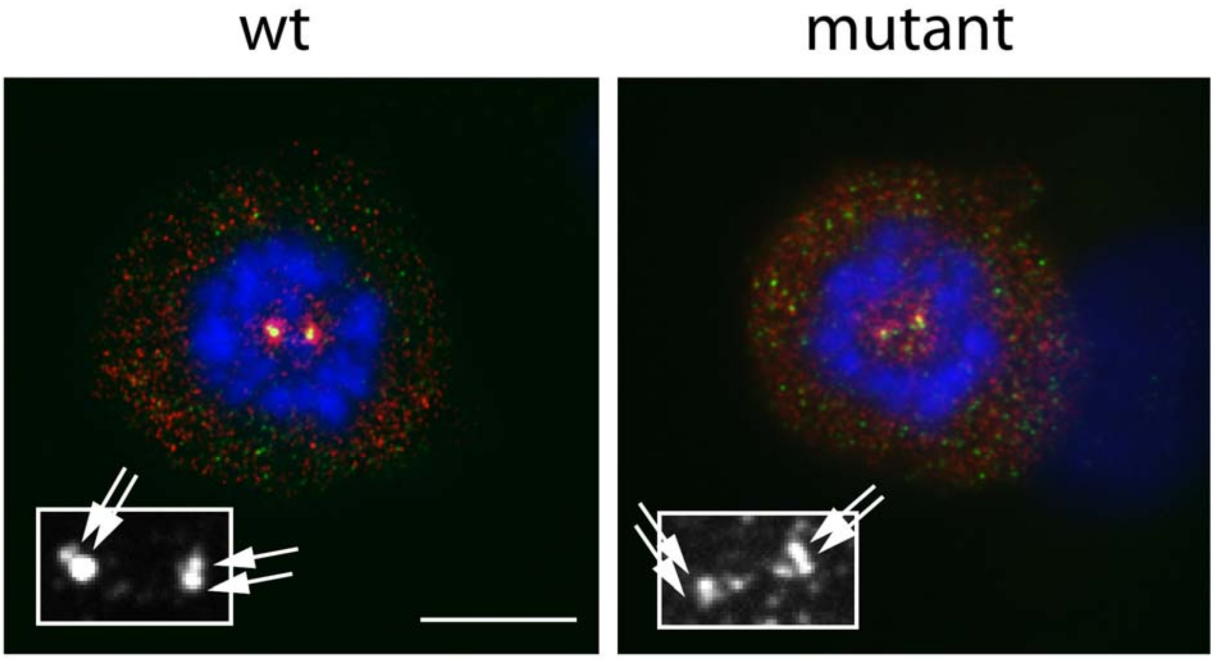

Supplementary Figure 3: Requirement of the mitotic kinesin Eg5 to assemble a bipolar mitotic spindle in SHGC mutant fibroblasts

Wild type and SHGC mutant primary fibroblasts were synchronized in G1/S with thymidine and released in the presence of monastrol for 8 hours. In both wild type and SHGC mutant cells, monastrol treatment inhibited Eg5-dependent centrosome separation in prophase, thereby arresting cells in mitosis with monopolar spindle with 2 pairs of centrioles. Centrioles were stained with GT335 antibody (green) and centrosomes with antibodies against gamma-tubulin (red). DNA was stained with DAPI (blue). Insets are enlargements of GT335 labeling to highlight the number of centrioles. White arrows point each centriole. Merge images are stacks of 4 sections of 0.2 µm each. Scale bar, 5 µm.

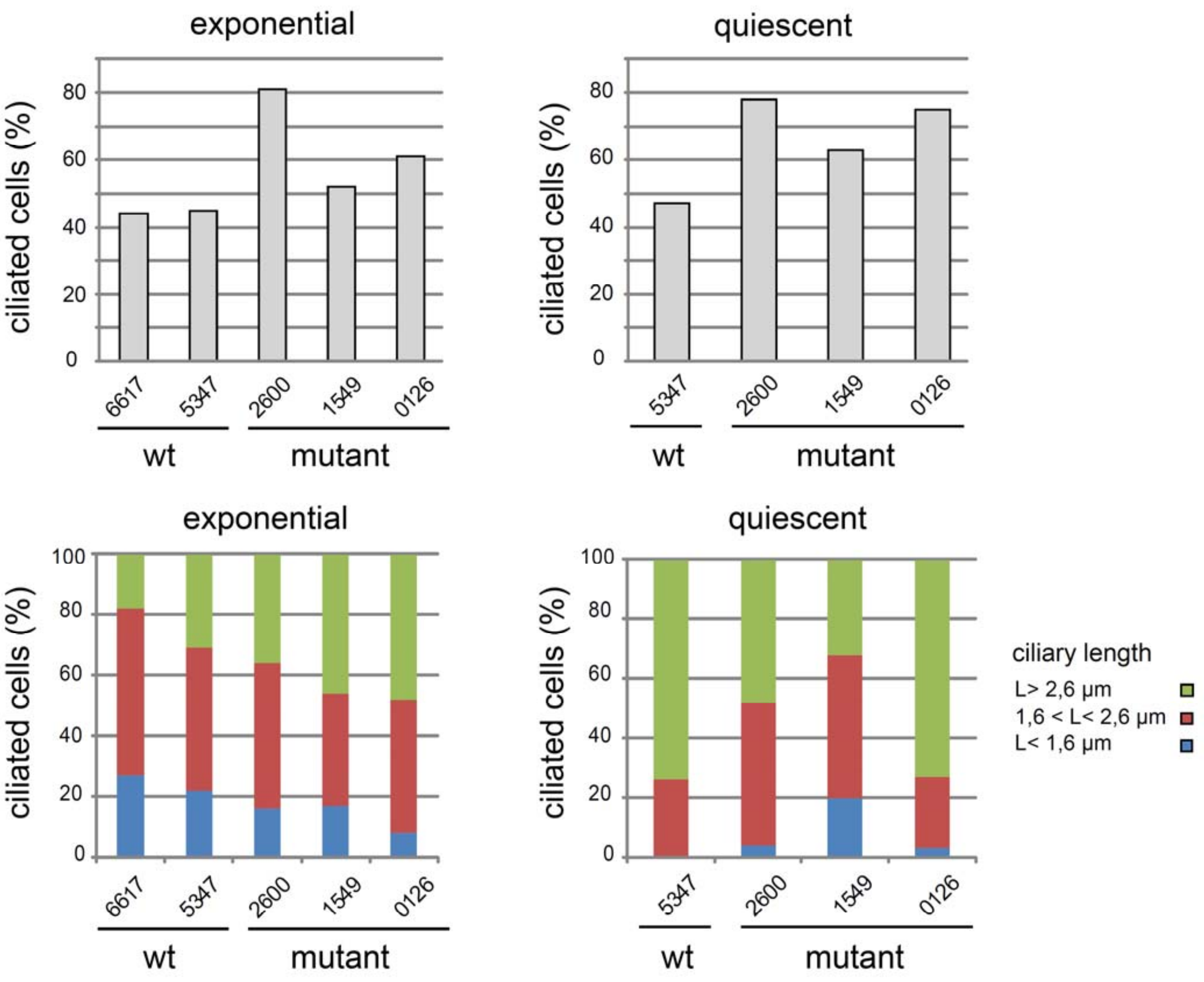

Supplementary Figure 4: Ciliogenesis in individual mutants

Upper panels show the percentage of ciliated cells during exponential growth on the left and in quiescent phase on the right. A cell was considered as ciliated when the length of the acetyl-tubulin staining was equal or longer than 0.4 µm.

Lower panels show ciliary length during exponential growth on the left and in quiescent phase on the right. For cells growing exponentially, n=164 cilia were measured for wild type cells (n=94 for animal 6617, n=70 for animal 5347) and n=388 cilia for SHGC mutant cells (n=95 for animal 2600, n=144 for animal 1549, n=149 for animal 0126). In quiescent cells arrested in G0 following high density and serum deprivation, n=124 cilia were measured for wild type cells (animal 5347) and n=400 cilia for SHGC mutant cells (n=93 for animal 2600, n=151 for animal 1549, n=156 for animal 0126). Cilia length was measured using ImageJ Software on Z-projections from different fields acquired with a SP5 Leica Confocal. Cilia length was not statistically different between wild type and mutant cells ($P>0.05$, Welch modified T test).

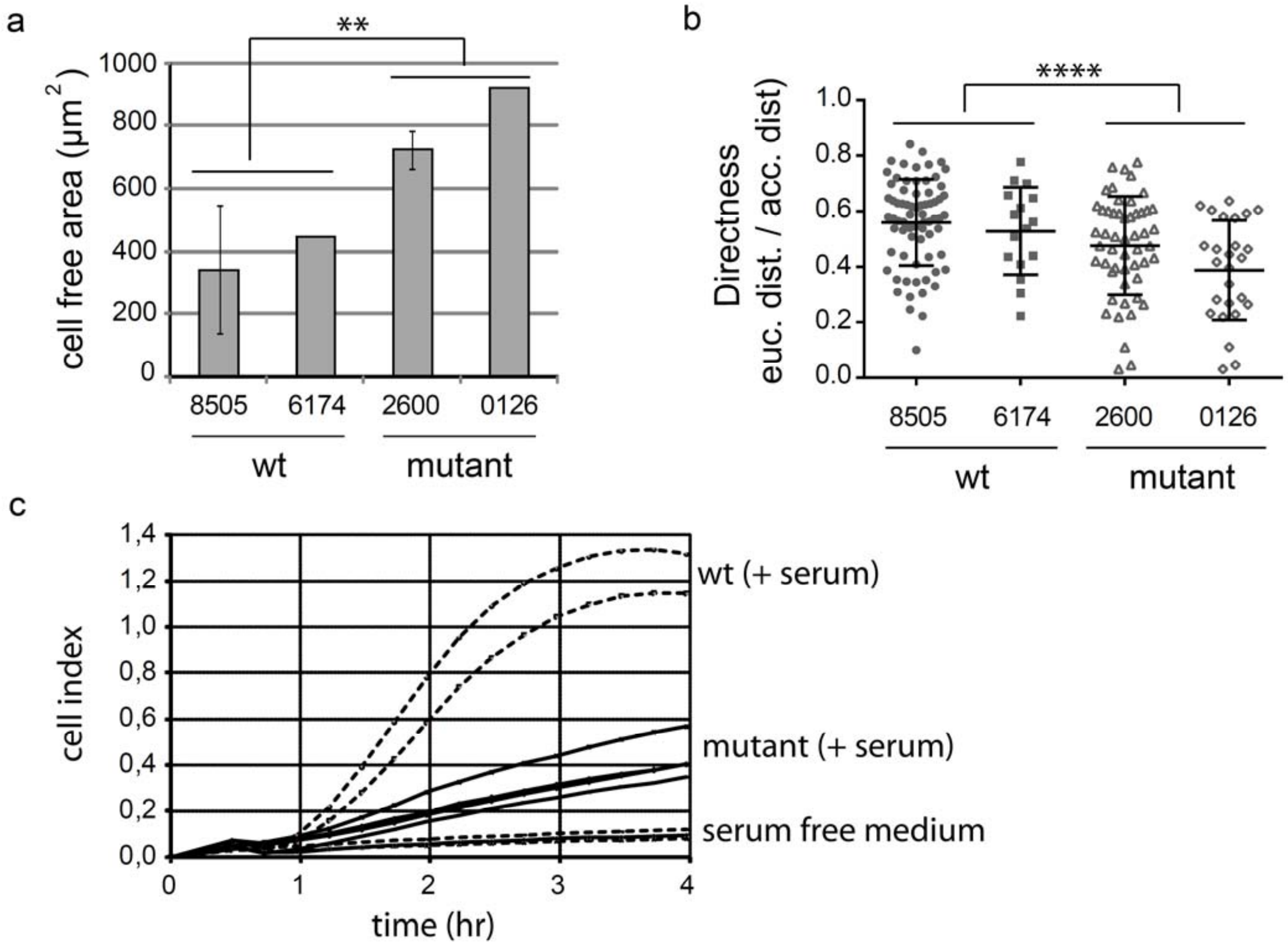

Supplementary Figure 5: Migration behavior in individual mutants

a. Wild type and SHGC mutant confluent quiescent fibroblasts migrated into the cell free gap of 500 µm during 20 hours (from wild type 8505 and 6174 cows versus SHCG mutant 2600 and 0126 cows). The cell free area was measured at 20 hours. Data shows mean and standard deviation from three independent experiments from wild type 8505 and SHCG mutant 2600. Values from one experiment with wild type 6174 and SHCG mutant 0126 fibroblasts are also represented.

b. The directionality was determined for each individual track, as the ratio of Euclidean distance/accumulated distance (directionality index). Data shows the mean, upper and lower decile of the distribution of values from three individual experiments in wild type 8505 or SHCG mutant 2600 fibroblasts, along with distribution of wild type 6174 or SHCG mutant 0126 cells tracks. The greater the directionality, the more linear the motion in a given direction is.

c. Directional migration was analyzed in real time using the xCELLigence platform. Wild type (dashed line) and SHGC mutant (continuous line) fibroblasts were seeded in the upper chamber in serum free medium and attracted to the bottom chamber with serum containing medium (+ serum) or in serum free medium as control. Graph shows modification of impedance (cell index) over time in a representative experiment out of 4, with fibroblasts from two different animals in each genotype.

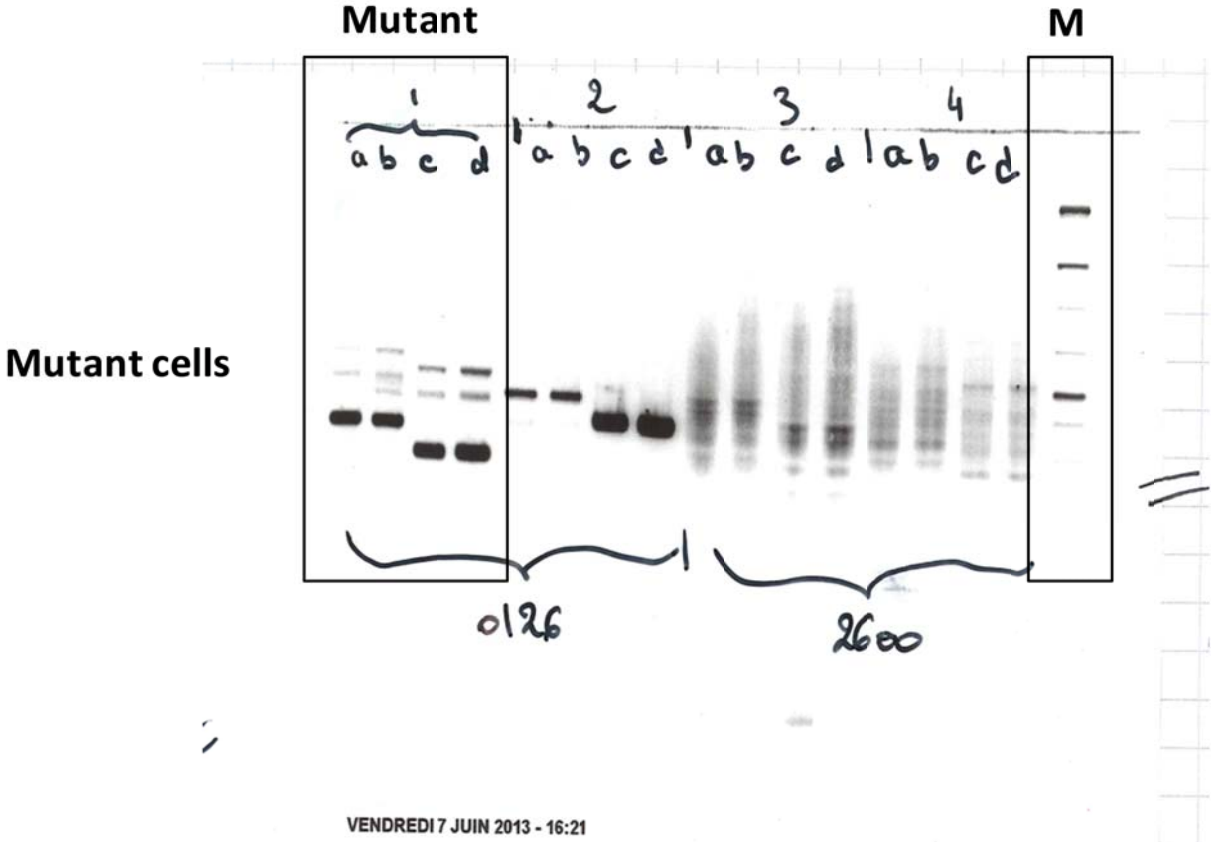

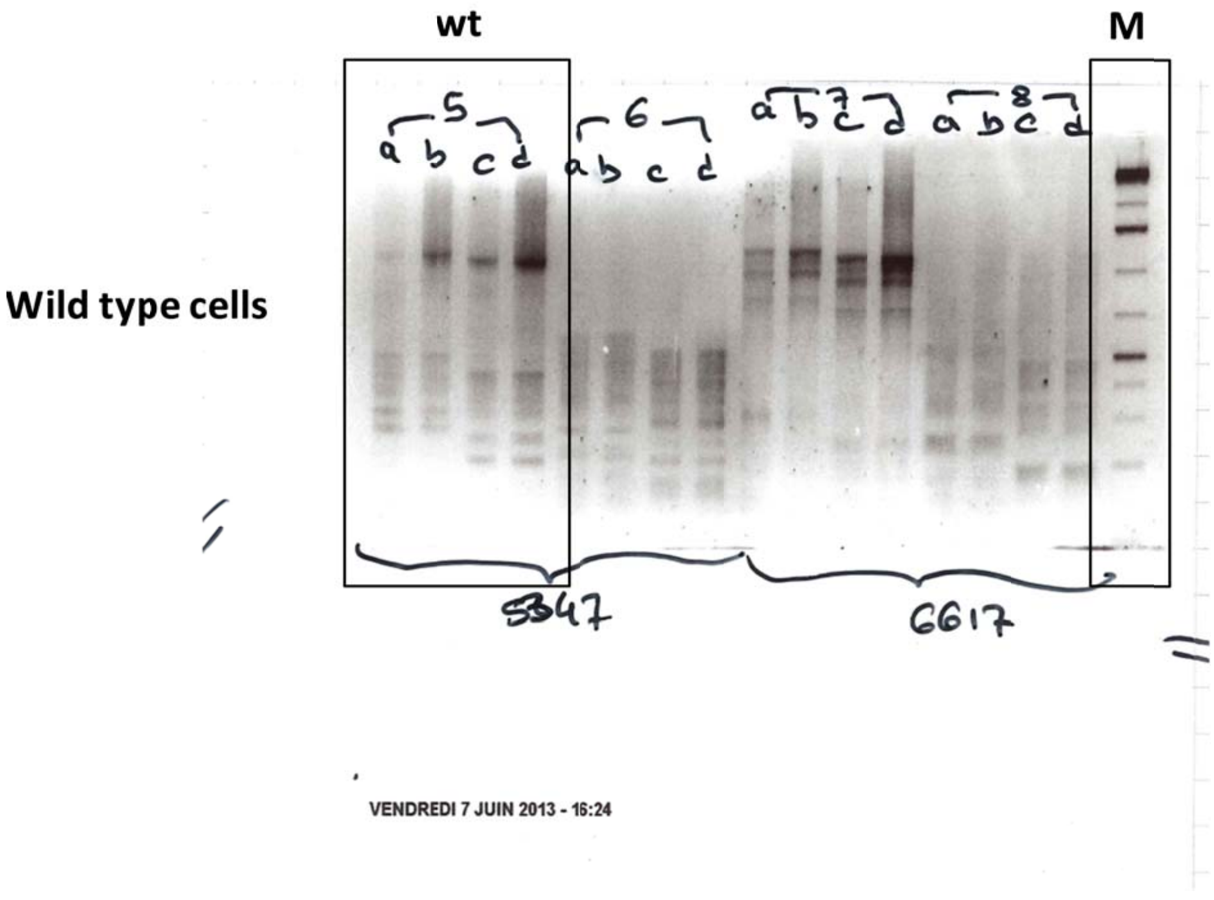

Supplementary Figure 6: Scan of the agarose gel presented on Figure 2b showing the 5’RACE products
Lanes used on Figure 2b are boxed.

Supplementary Table 1: Genetic markers and primer sequences used in the study

Linkage analysis

Name	Forward Primer (5' -> 3')	Reverse Primer (5' -> 3')
MS9	CCTACTGCCCTGAATTTCCTC	CCCAGGAAAGCCAGAAACTTA
MS113788	TCTCATGGAGAAGCCCAATC	CAGTCCATGGGGTCGATAAG
NMS3	ACACAGCTAGGCTGGATGCT	CCATAAGCCACGTAGTGCAG
MS386111	CCAGCAAGTCCTCATTCACA	CCCCAAATCATGACTGAACC
NMS28	CAGTCCCTCAGCCAGGATAG	AACAGTGACCCAACCACACA
MS2	TGCTATTCCTCTCTCCTCACG	GCAACAGGCAGAAAAGAAGTG
CEP250_MS	CGTGTGGGATTAAGCCTGAG	CAAGGGAAAAGAATCTGGAAAA

Name	Repeat motif	Amplicon positions on UMD_3.1 assembly
MS9	(TG)26(GA)14	chr13:63829843-63830081
MS113788	(TGC)12	chr13:63925821+63926076
NMS3	(TG)12	chr13:64278577-64278777
MS386111	(GT)13	chr13:64480499+64480642
NMS28	(TG)15	chr13:64572130+64572220
MS2	(CA)19	chr13:64611024-64611137
CEP250_MS	(TA)14	chr13:65400317+65400525

5' RACE

Name	Primers sequence(5' -> 3')
GSP1	GTCCAACTGCAAGGAGCTCTC
GSP2	GAACCTTGGGCCATACTGTCC
GSP3	GGGTTATATCCTGGATCACTTGC
GSP4	CCATTAATTCTGCATGATTTGCC

RT-qPCR

Name	Forward Primer	Reverse Primer
Exon2-Exon3	TTACAGTACCGGAGCTGGTG	TGTTCCAGGTTTGGCTTTTC
Exon4-Exon5	CGGGATGAGCTAATGAGGAA	TGGCTGACTTCATTTCCAGA
Exon5-Exon6	CTGGAAATGAAGTCAGCCACT	TGGTGGTACTCCCACAGTCA
Exon28-Exon29	CTGACCAGCCAAGCAGAAC	TCTCCTCTGCCTCAGGACTC
Exon30-Exon31	GGGTCCTAGAGAGCAGTGGA	GCATCCGAGGAGACTTTCTG

Supplementary Table 2: Mean genomic coverage obtained by FLX454 sequencing

Sample	11254 (healthy)	20892 (carrier)	20543 (SHGC)	20891 (SHGC)
Number of reads mapped on UMD3.1	102278	193209	153865	203717
Mean read length	316.8	286.8	318.3	281.8
Mean coverage (X)	6.2	10.6	9.4	11.0
